# Nitro­sonium complexation by the tetra­phospho­nate cavitand 5,11,17,23-tetra­methyl-6,10:12,16:18,22:24,4-tetra­kis­(phenyl­phospho­nato-κ^2^ *O*,*O*)resorcin(4)arene

**DOI:** 10.1107/S2056989017015857

**Published:** 2017-11-03

**Authors:** Roberta Pinalli, Chiara Massera

**Affiliations:** a Dipartimento di Scienze Chimiche, della Vita e della Sostenibilità Ambientale, Università di Parma, Parco Area delle Scienze 17/A, 43124 Parma, Italy

**Keywords:** crystal structure, tetra­phospho­nate cavitands, inclusion compounds, nitro­sonium ion, C—H⋯F inter­actions, C—H⋯π inter­actions

## Abstract

The crystal structure of a new supra­molecular complex between the tetra­phos­pho­nate cavitand 5,11,17,23-tetra­methyl-6,10:12,16:18,22:24,4-tetra­kis(phenyl­phospho­nato-κ^2^ *O*,*O*′)resorcin(4)arene and the nitrosyl cation NO^+^, as the BF_4_^−^ salt, is reported. The complex, of general formula [(C_56_H_44_P_4_O_12_)(NO)]BF_4_·CH_2_Cl_2_ or NO@Tiiii[H, CH_3_, C_6_H_5_] BF_4_·CH_2_Cl_2_, crystallizes in the space group *P*-1. The nitrosyl cation is disordered over two equivalent positions, with occupancies of 0.503 (2) and 0.497 (2), and inter­acts with two adjacent P=O groups at the upper rim of the cavitand through dipole–charge inter­actions. In the lattice, the cavitands are connected through a series of C—H⋯π inter­actions involving the methyl and methyl­enic H atoms and the aromatic rings of the macrocycle. The structure is further stabilized by the presence of C—H⋯F inter­actions between the hydrogen atoms of the cavitands and the F atoms of the tetra­fluorido­borate anion. As a result of the disorder, the lattice di­chloro­methane mol­ecules could not be modelled in terms of atomic sites, and were treated using the *PLATON* SQUEEZE procedure [Spek (2015 ▸). *Acta Cryst.* C**71**, 9–18]. The complexation process has also been studied in solution through NMR titrations.

## Abstract 2

Resorcinarene-based tetra­phospho­nate cavitands are versatile mol­ecular receptors which combine a π-basic aromatic cavity with hydrogen-bond acceptor groups at their upper rim. Their complexation properties span from neutral mol­ecules to cationic species, and have been extensively studied both in solution and in the solid state. In this paper, we report the NMR solution studies and the crystal structure of a new supra­molecular complex between a tetra­phospho­nate cavitand and the nitrosyl cation NO^+^. The cation is disordered over two equivalent positions, and inter­acts with two adjacent P=O groups at the upper rim of the cavitand through a dipole–charge inter­action.

## Chemical context   

Cavitands (Cram, 1983 ▸; Cram & Cram, 1994 ▸) are synthetic organic compounds endowed with a rigid, pre-organized cavity that have been used extensively both in solution (Hooley & Rebek, 2009 ▸; Pochorovski *et al.*, 2012 ▸) and in the solid state (Riboni *et al.*, 2016 ▸) as mol­ecular receptors for neutral mol­ecules and cationic species (Pinalli & Dalcanale, 2013 ▸). This versatility stems from the possibility of decorating both the upper and the lower rim of the resorcinarene skeleton with desired functionalities.

In our group, we have been particularly inter­ested in tetraphospho­nate cavitands of the general formula Tiiii[*R*, *R*_1_, *R*_2_] (*R* = lower rim substituents; *R*_1_ = upper rim substituents; *R*_2_ = substituents on the P atom) in which the upper rim of the macrocycle is functionalized with four P=O groups, all pointing inwards towards the cavity (Pinalli & Dalcanale, 2013 ▸). In this way, the π basicity of the cavity, useful for C—H⋯π recognition, is enriched with dipolar groups that can act both as hydrogen-bond acceptors and inter­act with cationic species through cation–dipole inter­actions.

The nitro­sonium ion and its salts have been studied in the past to investigate similarities and differences with the O_2_^+^ ion in terms of size, ionization potential, electron affinity, oxidation power *etc* (Mazej *et al.*, 2009 ▸). Moreover, the NO^+^ cation can be used as a model for nitro­gen oxides in mol­ecular recognition phenomena. Indeed, the formation of stable, host–guest complexes between NO^+^ cations and organic mol­ecular receptors has been studied in solution with resorcinarenes (Botta *et al.*, 2007 ▸) or with calixarenes, both in solution (Zyryanov *et al.*, 2002 ▸, 2003 ▸) and in the solid state (Rathore *et al.*, 2000 ▸). In particular, nitro­sonium hexa­chloro­anti­monate was shown to form an inclusion compound with tetra­meth­oxy- and tetra-*n*-propoxycalix(4)arenes due to the inter­action between the positive charge of the guest and the electron-rich aromatic cavity of the host (Rathore *et al.*, 2000 ▸). Inspired by this work, we decided to carry out a combined solution and solid-state study of the complexation properties of the rigid tetra­phospho­nate cavitand 5,11,17,23-tetra­methyl-6,10:12,16:18,22:24,4-tetra­kis­(phenyl­phospho­nato-*O*,*O*′)res­orcin(4)arene (from now on indicated as Tiiii[H, CH_3_, C_6_H_5_]) towards NOBF_4_.
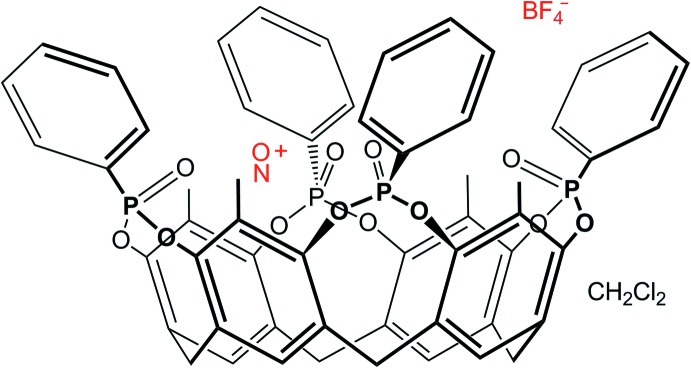


## Studies in solution   

Preliminary ^31^P and ^1^H NMR studies were performed to probe the complexation properties of the cavitand towards the nitro­sonium ion in solution. To this purpose, we synthesized the cavitand Tiiii[C_3_H_7_, CH_3_, C_6_H_5_], functionalized at the lower rim with four –C_3_H_7_ alkyl chains to enhance the cavitand solubility. The NMR tube was filled with 0.5 ml of a CDCl_3_ solution containing the cavitand (1 mmol concentration). The NOBF_4_ titrant solution was prepared by dissolving the guest in 0.4 ml (10 mmol) of the above-mentioned cavitand solution to keep the concentration of the host constant during the titration. Portions (0.25 eq., 22.5 µL) of the titrant were added by syringe to the NMR tube. During the titration, the phospho­rous singlet of the cavitand shifted slightly downfield, from 6.01 (signal for the free host) to 7.42 ppm upon addition of an excess (2.5 eq.) of the guest (see Fig. S1 in the *Supporting information*), indicating the presence of cation–dipole inter­actions between the nitro­sonium ion and the phospho­nate groups at the upper rim. The broadening of the signal is due to the fast exchange (at the NMR time scale) of the guest inside the cavity.

**Figure 1:**
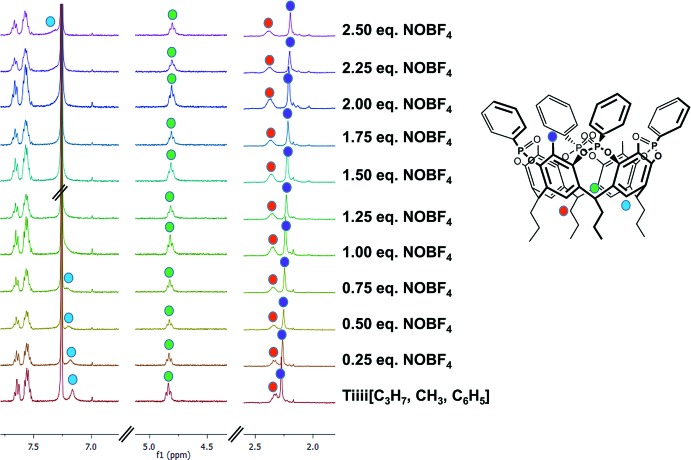
Selected portions of the ^1^H NMR (400 MHz, CDCl_3_, 298 K) spectra recorded during the titration of the cavitand with increasing equivalents of NOBF_4_.

In Fig. 1 ▸, the comparison between the ^1^H spectra recorded after each guest addition is reported. As can be seen, the protons of the methyl group in the apical position of the cavitand skeleton (purple dot) are shifted up-field, increasing the guest concentration; this means that the presence of the NO^+^ cation in proximity to the cavitand upper rim creates a change in the environment, which results in an overall shielding effect. On the contrary, the signals of the protons at the lower rim, namely the aromatic hydrogens (light-blue dot), the bridging methines (green dot) and the alkyl methylenic groups (red dot), are shifted downfield. This is due to the perturbation created by the BF_4_^−^ anion, which is likely positioned among the alkyl feet of the cavitand, as already observed for counter-anions in other crystal structures previously reported (Pinalli *et al.*, 2016 ▸). Also in this case, broadening of the signals was observed.

Following these results, solid-state studies were carried out to obtain an insight into the type, number, strength and geometry of the weak inter­actions taking place in the system.

## Structural commentary   

**Figure 2:**
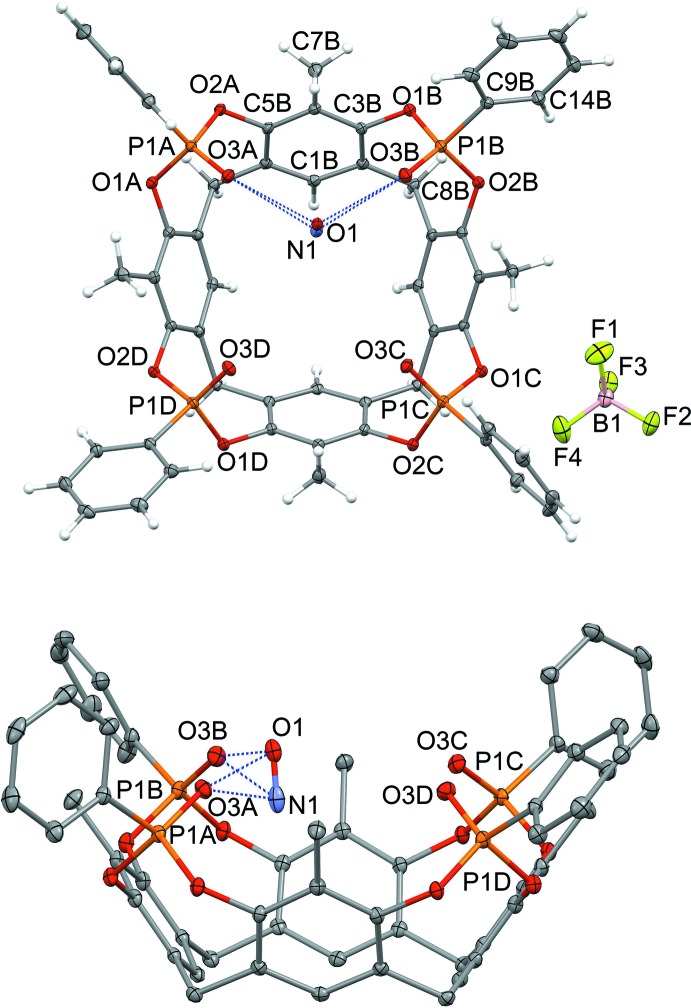
Top and side views of the title compound, NO@Tiiii[H, CH_3_, C_6_H_5_], with a partial atom-labelling scheme. Displacement ellipsoids are drawn at the 20% probability level. Only one of the two disordered NO^+^ ions is shown. In the side view, the hydrogen atoms and the BF_4_^−^ counter-ion are not shown for clarity. Cation–dipole inter­actions are represented as blue dashed lines.

The mol­ecular structure of NO@Tiiii[H, CH_3_, C_6_H_5_]BF_4_·CH_2_Cl_2_ is reported in Fig. 2 ▸. The complex crystallizes in the space group *P* 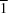, and the asymmetric unit comprises one cavitand, one mol­ecule of NOBF_4_ (with the cation disordered over two equivalent positions) and one disordered mol­ecule of di­chloro­methane. The NO^+^BF_4_^−^ ionic pair is separated, and the nitro­sonium ion is located within the macrocycle, not deep inside the cavity, but lying in the mean plane passing through the four phospho­nate oxygen atoms O3*A*, O3*B*, O3*C* and O3*D* (for detailed geometrical parameters, see Table 1 ▸). The nitro­gen and oxygen atoms of the guest point towards the lower and the upper rims, respectively, and are held in place *via* cation–dipole inter­actions with two adjacent P=O groups. It is inter­esting to note that the NO^+^ ion is disordered with 50% probability over two equivalent orientations [N1O1 with occupancy of 0.503 (2) and N2O2 with occupancy of 0.497 (2)], thus forming alternately an inter­action with each of the two opposite P=O groups (Fig. 2 ▸; the second orientation is not shown), namely P1*A*=O3*A* and P1*B*=O3*B* for N1O1 and P1*C*=O3*C* and P1*D*=O3*D* with N2O2 [O3*A*⋯O1, 2.621 (5); O3*A*⋯N1, 2.661 (6); O3*B*⋯O1, 2.609 (3); O3*B*⋯N1, 2.664 (5); O3*C*⋯O2, 2.621 (4); O3*C*⋯N2, 2.625 (7); O3*D*⋯O2, 2.604 (4); O3*D*⋯N2, 2.650 (4) Å]. This phenomenon has already been observed in the solid state with phospho­nate cavitands hosting methanol and ethanol mol­ecules (Melegari *et al.*, 2008 ▸) and confirms that, for these systems, the stability of the host–guest complex is entropic in origin, since the guest can choose from two up to four energetically and geometrically equivalent inter­action modes with the host. In this case, the NO^+^ cation forms two sets of strong inter­actions with two adjacent P=O groups, which results in a better stabilizing effect than four weaker inter­actions with all the phospho­nate moieties of the upper rim. The BF_4_^−^ ion is outside the cavity, forming weak C—H⋯F inter­actions with the cavitands (see *Section 4* for details).

The di­chloro­methane solvent mol­ecule is heavily disordered and could not be modelled, but its residual electron density, occupying a void of 312 Å^3^ (Spek, 2015 ▸) is located in the hydro­phobic pockets among the cavitands.

## Supra­molecular features   

**Figure 3:**
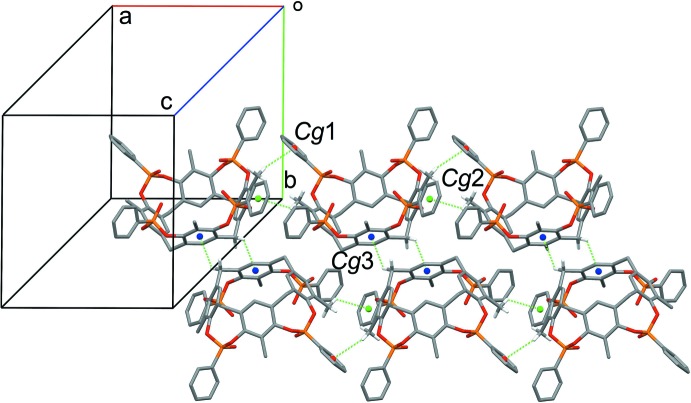
C—H⋯π inter­actions (green dashed lines) forming a ribbon along the *a-*axis direction of the unit cell. Centroids *Cg*1 (C9*B*–C14*B*), *Cg*2 (C9*D*–C14*D*) and *Cg*3 (C1*A*–C6*A*) are represented as red, green and blue spheres, respectively.

**Figure 4:**
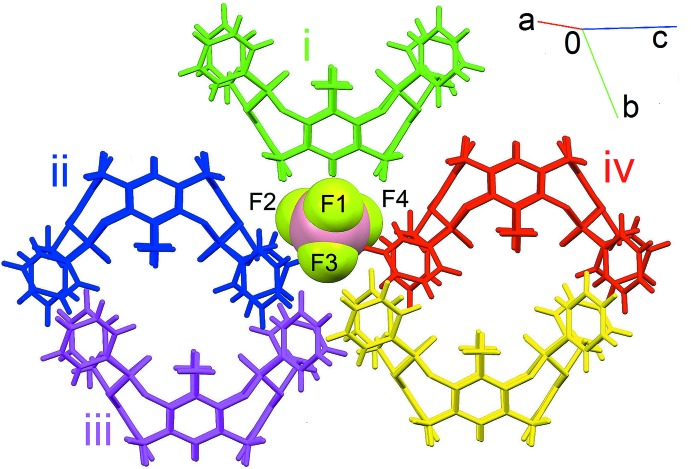
View of the BF_4_^−^ ion surrounded by the five closest cavitands through C—H⋯F inter­actions. [Symmetry codes: (i) *x*, *y* − 1, *z*; (ii) −*x* + 1, −*y* + 1, −*z*; (iii) *x*, *y*, *z* − 1; (iv) −*x*, −*y* + 1, −*z* + 1.]

In the lattice, the cavitands form a supra­molecular ribbon along the *a*-axis direction through a series of C—H⋯π inter­actions between the H atoms of the methyl groups at the upper rim and the phenyl rings of the phospho­nato moieties. In particular, each cavitand inter­acts with two adjacent ones acting simultaneously as a donor to two methyl groups and as an acceptor to two aromatic rings (see Table 2 ▸ and Fig. 3 ▸; the centroids involved are *Cg*1 and *Cg*2, represented as red and green spheres, respectively). Moreover, pairs of centrosymmetric cavitands form another set of C—H⋯π inter­actions involving the methyl­enic hydrogen atoms at the lower rim and the aromatic walls of the macrocycle (see Table 2 ▸ and Fig. 3 ▸, *Cg*3, blue centroids). The structure is further stabilized by the presence of C—H⋯F inter­actions between the hydrogen atoms of the cavitands and the fluorine atoms of the tetra­fluorido­borate anion. More precisely, each BF_4_^−^ is surrounded by five cavitands (Fig. 4 ▸), with C—H⋯F distances ranging from 2.408 (2) to 2.653 (2) Å (Table 2 ▸).

## Database survey   

**Figure 5:**
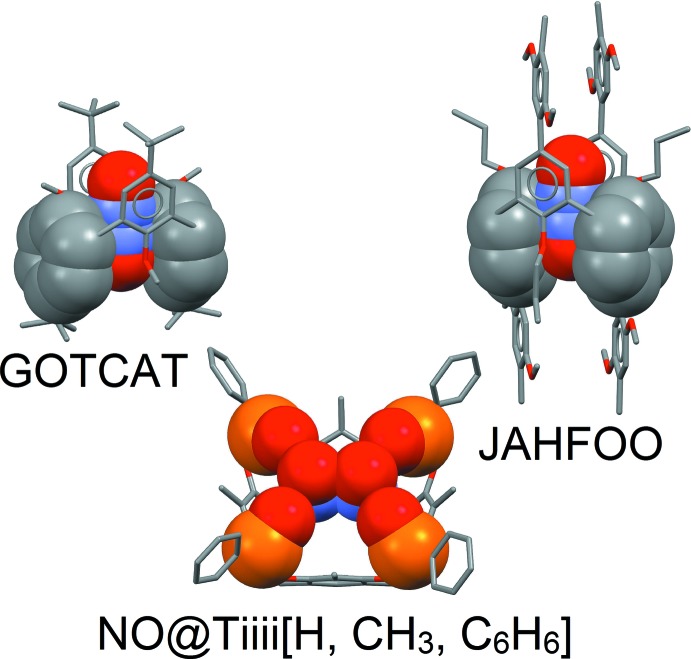
Comparison of the inter­action modes of GOTCAT, JAHFOO (side view), and of the title compound, NO@Tiiii[H, CH_3_, C_6_H_5_] (top view), highlighting the disorder of the guest over two equivalent positions. The space-filling view is only partial for reasons of clarity.

A search in the Cambridge Structural Database (Version 5.38, update May 2017; Groom *et al.*, 2016 ▸) for structures containing the isolated NO fragment, with no restrictions on the charge or on the type of bond connecting nitro­gen and oxygen, yielded 65 species which are, of course, very different in nature. Meaningful comparisons with our complex are only possible with the series of calixarene-based, host–guest complexes already cited in the introduction, namely GOTCAT, GOTDEY, GOTGEB, GOTHAY and GOTHAY01 (Rathore *et al.*, 2000 ▸) and with a cationic radical calixarene derivative capable of binding neutral nitric oxide (JAHFOO; Rathore *et al.*, 2004 ▸). In particular, in GOTCAT, the NO^+^ cation is buried deep inside the cavity, where it inter­acts with two distal aromatic groups of the calixarene guest. Since the calixarene is in the 1,3-alternate conformation, two sets of co-facial benzene rings are present, and the NO^+^ ion is equally distributed between them (see Fig. 5 ▸, one pair of rings is shown in space-filling model, the other one in capped-stick mode). The electron-rich pocket formed by the co-facial pair is essential for the complexation, and the NO^+^ ion is not bound by a single aromatic ring alone (see, for instance, GOTDEY and GOTGEB). In the case of JAHFOO, the calixarene has been oxidized to carry an overall positive charge on its core, in order to make it a good receptor for an electron rich-guest such as nitric oxide. Nevertheless, the inter­action mode is similar to that observed for GOTCAT, with two disordered NO mol­ecules buried between two distinct pairs of distal aromatic rings (Fig. 5 ▸). Also, in the title complex the guest is disordered over two equivalent positions, but its inter­action with the electron-rich cavity is negligible due to the presence of the dipolar phospho­nate groups which ‘hold’ the NO^+^ ion at the brim of the upper rim (Fig. 5 ▸).

## Synthesis and crystallization   

^1^H NMR spectra were obtained using a Bruker AMX-400 (400 MHz) spectrometer. All chemical shifts (δ) were reported in ppm relative to the proton resonances resulting from incomplete deuteration of the NMR solvents. ^31^P NMR spectra were obtained using a Bruker AMX-400 (162 MHz) spectrometer. All chemical shifts (δ) were recorded in ppm relative to external 85% H_3_PO_4_ at 0.00 ppm. All commercial reagents were ACS reagent grade and used as received. The cavitands Tiiii[H, CH_3_, C_6_H_5_] and Tiiii[C_3_H_7_, CH_3_, C_6_H_5_] were prepared following published procedures (Tonezzer *et al.*, 2008 ▸; Menozzi *et al.*, 2015 ▸).

NO@Tiiii[H, CH_3_, C_6_H_5_]BF_4_·CH_2_Cl_2_ was obtained by mixing a di­chloro­methane solution of Tiiii[H, CH_3_, C_6_H_5_] (1 eq.) with a di­chloro­methane solution of NOBF_4_ (1 eq.). The mixture was left to evaporate to yield colourless single crystals of the 1:1 complex that were suitable for X-ray diffraction analysis.

## Refinement   

Crystal data, data collection and structure refinement details are summarized in Table 3 ▸. The nitro­sonium ion was found to be disordered over two positions, with a refined occupancy ratio of 0.503 (2):0.497 (2). The C-bound H atoms were placed in calculated positions and refined using a riding model: C—H = 0.95-0.98 Å with *U*_iso_(H) = 1.5*U*_eq_(C-meth­yl) and 1.2*U*_eq_(C) for other H atoms.

As a result of severe disorder, the CH_2_Cl_2_ solvent could not be sensibly modelled in terms of atomic sites, and was treated using the *PLATON* SQUEEZE procedure (Spek, 2015 ▸); the solvent contribution to the diffraction pattern was removed and modified *F*_o_^2^ written to a new HKL file. The number of electrons corresponding to the solvent mol­ecules were included in the formula, formula weight, calculated density, μ and *F*(000).

## Supplementary Material

Crystal structure: contains datablock(s) I, Global. DOI: 10.1107/S2056989017015857/su5400sup1.cif

Structure factors: contains datablock(s) I. DOI: 10.1107/S2056989017015857/su5400Isup2.hkl

Figure showing the 31P NMR (162 MHz, CDCl3, 298 K) spectra recorded during the titration of the cavitand with increasing equivalents of NOBF4.. DOI: 10.1107/S2056989017015857/su5400sup3.tif

CCDC reference: 1583086

Additional supporting information:  crystallographic information; 3D view; checkCIF report

## Figures and Tables

**Table 1:** Host–guest inter­actions (Å) in NO@Tiiii[H, CH_3_, C_6_H_5_]BF_4_

O3*A*···O1	2.621 (5)	O3*D*···O2	2.604 (4)
O3*A*···N1	2.661 (6)	O3*D*···N2	2.650 (4)
O3*B*···O1	2.609 (3)	O1···PL	0.471 (4)
O3*B*···N1	2.664 (5)	N1···PL	0.492 (6)
O3*C*···O2	2.621 (4)	O2···PL	0.466 (4)
O3*C*···N2	2.625 (7)	N2···PL	0.416 (6)

PL is the mean plane passing through the four phospho­nate oxygen atoms, O3*A*, O3*B*, O3*C* and O3*D*.

**Table 2:** Hydrogen-bond geometry (Å, °) *Cg*1, *Cg*2 and *Cg*3 are the centroids of the aromatic rings C9*B*–C14*B*, C9*D*–C14*D* and C1*A*–C6*A*, respectively.

*D*—H⋯*A*	*D*—H	H⋯*A*	*D*⋯*A*	*D*—H⋯*A*
C1*B* ^i^—H1*B* ^i^⋯F1	0.95	2.41	3.344 (3)	169
C14*B* ^ii^—H14*B* ^ii^⋯F2	0.95	2.57	3.357 (3)	140
C7*C* ^ii^—H7*C*3^ii^⋯F2	0.98	2.62	3.484 (2)	147
C8*C* ^i^—H8*C*1^i^⋯F2	0.98	2.49	3.379 (3)	150
C1*D* ^i^—H1*D* ^i^⋯F2	0.95	2.60	3.439 (2)	147
C11*A* ^iii^—H11*A* ^iii^⋯F3	0.95	2.45	3.254 (2)	142
C7*C* ^ii^—H7*C*3^ii^⋯F3	0.98	2.64	3.569 (3)	160
C11*C*—H11*C*⋯F4	0.95	2.53	3.447 (3)	162
C1*D* ^i^—H1*D* ^i^⋯F4	0.95	2.65	3.509 (3)	150
C14*D* ^iv^—H14*D* ^iv^⋯F4	0.95	2.63	3.336 (4)	131
C7*D*—H7*D*1⋯*Cg*1^v^	0.98	2.80	3.524 (4)	131
C7*B*—H7*B*1⋯*Cg*2^vi^	0.98	2.88	3.530 (4)	124
C8*D*—H8*D*2⋯*Cg*3^vii^	0.98	2.87	3.594 (3)	131

Symmetry codes: (i) 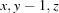; (ii) 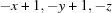; (iii) 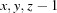; (iv) 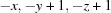; (v) 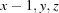; (vi) 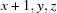; (vii) 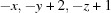.

**Table 3:** Experimental details

Crystal data	
Chemical formula	C_56_H_44_P_4_O_12_·NO^+^·BF_4_^−^·CH_2_Cl_2_
*M*_r_	1234.54
Crystal system, space group	Triclinic, *P* 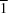
Temperature (K)	190
*a*, *b*, *c* (Å)	13.856 (1), 14.909 (2), 16.357 (2)
α, β, γ (°)	63.224 (2), 73.137 (2), 88.093 (2)
*V* (Å^3^)	2868.2 (6)
*Z*	2
Radiation type	Mo *K*α
μ (mm^−1^)	0.30
Crystal size (mm)	0.16 × 0.13 × 0.10

Data collection	
Diffractometer	Bruker SMART BREEZE CCD area-detector
Absorption correction	Multi-scan (*SADABS*; Bruker, 2008 ▸)
*T*_min_, *T*_max_	0.812, 1.000
No. of measured, independent and observed [*I* > 2σ(*I*)] reflections	36384, 14109, 9478
*R*_int_	0.033
(sin θ/λ)_max_ (Å^−1^)	0.690

Refinement	
*R*[*F* ^2^ > 2σ(*F* ^2^)], *wR*(*F* ^2^), *S*	0.042, 0.128, 1.00
No. of reflections	14109
No. of parameters	735
H-atom treatment	H-atom parameters constrained
Δρ_max_, Δρ_min_ (e Å^−3^)	0.63, −0.41

Computer programs: *APEX2* and *SAINT* (Bruker, 2008 ▸), *SIR97* (Altomare *et al.*, 1999 ▸), *SHELXL2014* (Sheldrick, 2015 ▸), *Mercury* (Macrae *et al.*, 2008 ▸), *WinGX* (Farrugia, 2012 ▸), *PARST* (Nardelli, 1995 ▸) and *publCIF* (Westrip, 2010 ▸).

## supplementary crystallographic information

### Crystal data

**Table:**

C_56_H_44_P_4_O_12_·NO^+^·BF_4_^−^·CH_2_Cl_2_	*Z* = 2
*M**_r_* = 1234.54	*F*(000) = 1268
Triclinic, *P*1	*D*_x_ = 1.429 Mg m^−^^3^
*a* = 13.856 (1) Å	Mo *K*α radiation, λ = 0.71069 Å
*b* = 14.909 (2) Å	Cell parameters from 250 reflections
*c* = 16.357 (2) Å	θ = 1.5–29.4°
α = 63.224 (2)°	µ = 0.30 mm^−^^1^
β = 73.137 (2)°	*T* = 190 K
γ = 88.093 (2)°	Prismatic, colourless
*V* = 2868.2 (6) Å^3^	0.16 × 0.13 × 0.10 mm

### Data collection

**Table:**

Bruker SMART BREEZE CCD area-detector diffractometer	14109 independent reflections
Radiation source: fine-focus sealed tube	9478 reflections with *I* > 2σ(*I*)
Graphite monochromator	*R*_int_ = 0.033
ω scan	θ_max_ = 29.4°, θ_min_ = 1.5°
Absorption correction: multi-scan (SADABS; Bruker, 2008)	*h* = −18→18
*T*_min_ = 0.812, *T*_max_ = 1.000	*k* = −20→20
36384 measured reflections	*l* = −22→22

### Refinement

**Table:**

Refinement on *F*^2^	Primary atom site location: structure-invariant direct methods
Least-squares matrix: full	Secondary atom site location: difference Fourier map
*R*[*F*^2^ > 2σ(*F*^2^)] = 0.042	Hydrogen site location: inferred from neighbouring sites
*wR*(*F*^2^) = 0.128	H-atom parameters constrained
*S* = 1.00	*w* = 1/[σ^2^(*F*_o_^2^) + (0.0693*P*)^2^] where *P* = (*F*_o_^2^ + 2*F*_c_^2^)/3
14109 reflections	(Δ/σ)_max_ = 0.001
735 parameters	Δρ_max_ = 0.63 e Å^−^^3^
0 restraints	Δρ_min_ = −0.41 e Å^−^^3^

### Special details

**Table:**

Experimental. The calculated molar mass, density and absorption coefficient include two disordered dichloromethane molecules per cell which do not appear in the final files because of the refinements carried out with data subjected to SQUEEZE.
Geometry. All esds (except the esd in the dihedral angle between two l.s. planes) are estimated using the full covariance matrix. The cell esds are taken into account individually in the estimation of esds in distances, angles and torsion angles; correlations between esds in cell parameters are only used when they are defined by crystal symmetry. An approximate (isotropic) treatment of cell esds is used for estimating esds involving l.s. planes.

### Fractional atomic coordinates and isotropic or equivalent isotropic displacement parameters (Å^2^)

**Table:**

	*x*	*y*	*z*	*U*_iso_*/*U*_eq_	Occ. (<1)
N1	0.3415 (3)	0.7159 (4)	0.4179 (3)	0.0386 (10)	0.503 (2)
O1	0.3391 (2)	0.6435 (3)	0.4543 (2)	0.0307 (8)	0.503 (2)
N2	0.2020 (3)	0.7031 (4)	0.3846 (3)	0.0426 (11)	0.497 (2)
O2	0.1903 (2)	0.6363 (3)	0.4165 (2)	0.0266 (7)	0.497 (2)
B1	0.2882 (2)	0.2304 (2)	0.12591 (19)	0.0410 (6)	
F1	0.34181 (17)	0.16872 (13)	0.18534 (15)	0.0875 (6)	
F2	0.28773 (14)	0.19718 (12)	0.05981 (11)	0.0638 (4)	
F3	0.33203 (12)	0.32872 (10)	0.08112 (10)	0.0553 (4)	
F4	0.19008 (14)	0.22111 (13)	0.18451 (14)	0.0790 (6)	
P1A	0.35319 (4)	0.75850 (4)	0.63016 (3)	0.02411 (12)	
P1B	0.60883 (4)	0.73524 (4)	0.25305 (4)	0.02623 (12)	
P1C	0.19872 (4)	0.71498 (4)	0.14576 (3)	0.02352 (11)	
P1D	−0.05960 (4)	0.73655 (4)	0.52317 (4)	0.02430 (12)	
O1A	0.26652 (10)	0.82864 (10)	0.64092 (9)	0.0264 (3)	
O2A	0.45495 (10)	0.83412 (10)	0.56545 (9)	0.0256 (3)	
O3A	0.33168 (11)	0.69279 (11)	0.59071 (10)	0.0315 (3)	
O1B	0.64868 (10)	0.81454 (11)	0.27975 (10)	0.0286 (3)	
O2B	0.60145 (10)	0.80000 (11)	0.14780 (9)	0.0275 (3)	
O3B	0.51418 (10)	0.67359 (11)	0.32481 (10)	0.0344 (3)	
O1C	0.29952 (10)	0.78374 (10)	0.06637 (9)	0.0250 (3)	
O2C	0.11212 (10)	0.78858 (10)	0.13782 (9)	0.0260 (3)	
O3C	0.20873 (11)	0.66232 (11)	0.24296 (10)	0.0310 (3)	
O1D	−0.08197 (10)	0.80541 (10)	0.42444 (9)	0.0265 (3)	
O2D	−0.03700 (9)	0.81178 (10)	0.56143 (9)	0.0251 (3)	
O3D	0.02187 (10)	0.67259 (11)	0.51314 (10)	0.0331 (3)	
C1A	0.19513 (14)	0.99115 (14)	0.42444 (13)	0.0239 (4)	
H1A	0.2222	1.0501	0.3648	0.029*	
C2A	0.25267 (14)	0.95390 (14)	0.48748 (13)	0.0233 (4)	
C3A	0.21105 (14)	0.86730 (15)	0.57387 (13)	0.0239 (4)	
C4A	0.11502 (14)	0.81716 (14)	0.60086 (14)	0.0246 (4)	
C5A	0.06242 (14)	0.85866 (14)	0.53393 (13)	0.0235 (4)	
C6A	0.09877 (14)	0.94465 (14)	0.44598 (13)	0.0227 (4)	
C7A	0.07040 (16)	0.72542 (16)	0.69608 (15)	0.0345 (5)	
H7A1	0.1199	0.7067	0.7317	0.052*	
H7A2	0.0534	0.6692	0.6853	0.052*	
H7A3	0.0088	0.7405	0.7334	0.052*	
C8A	0.35871 (14)	1.00412 (15)	0.46023 (14)	0.0253 (4)	
H8A1	0.3721	0.9976	0.5188	0.030*	
H8A2	0.3630	1.0771	0.4160	0.030*	
C9A	0.36897 (14)	0.69707 (15)	0.74569 (14)	0.0265 (4)	
C10A	0.35124 (16)	0.59170 (16)	0.79585 (15)	0.0338 (5)	
H10A	0.3299	0.5545	0.7686	0.041*	
C11A	0.36511 (18)	0.54167 (19)	0.88606 (16)	0.0443 (6)	
H11A	0.3528	0.4700	0.9208	0.053*	
C12A	0.39638 (18)	0.5952 (2)	0.92518 (16)	0.0463 (6)	
H12A	0.4063	0.5604	0.9866	0.056*	
C13A	0.41351 (19)	0.6994 (2)	0.87595 (17)	0.0473 (6)	
H13A	0.4349	0.7360	0.9037	0.057*	
C14A	0.39949 (17)	0.75105 (18)	0.78587 (15)	0.0378 (5)	
H14A	0.4108	0.8228	0.7522	0.045*	
C1B	0.46749 (14)	0.99163 (14)	0.31262 (13)	0.0244 (4)	
H1B	0.4392	1.0488	0.2751	0.029*	
C2B	0.53674 (14)	0.94606 (14)	0.26635 (13)	0.0241 (4)	
C3B	0.57720 (14)	0.86308 (15)	0.32371 (14)	0.0250 (4)	
C4B	0.55347 (14)	0.82460 (15)	0.42299 (14)	0.0263 (4)	
C5B	0.48240 (14)	0.87296 (15)	0.46416 (13)	0.0246 (4)	
C6B	0.43862 (14)	0.95615 (14)	0.41178 (13)	0.0232 (4)	
C7B	0.60064 (17)	0.73656 (17)	0.48175 (15)	0.0365 (5)	
H7B1	0.6048	0.7425	0.5379	0.055*	
H7B2	0.6690	0.7360	0.4425	0.055*	
H7B3	0.5590	0.6735	0.5033	0.055*	
C8B	0.56298 (15)	0.98464 (15)	0.15868 (13)	0.0267 (4)	
H8B1	0.6338	0.9735	0.1331	0.032*	
H8B2	0.5584	1.0583	0.1275	0.032*	
C9B	0.71418 (15)	0.66821 (16)	0.23315 (15)	0.0296 (4)	
C10B	0.72732 (17)	0.58210 (17)	0.31116 (18)	0.0373 (5)	
H10B	0.6810	0.5605	0.3739	0.045*	
C11B	0.80909 (18)	0.52802 (18)	0.2962 (2)	0.0459 (6)	
H11B	0.8188	0.4697	0.3489	0.055*	
C12B	0.87546 (18)	0.5590 (2)	0.2052 (2)	0.0470 (6)	
H12B	0.9313	0.5223	0.1955	0.056*	
C13B	0.86170 (18)	0.6421 (2)	0.1286 (2)	0.0497 (7)	
H13B	0.9075	0.6620	0.0659	0.060*	
C14B	0.78131 (17)	0.6980 (2)	0.14123 (17)	0.0434 (6)	
H14B	0.7724	0.7560	0.0877	0.052*	
C1C	0.40529 (14)	0.97320 (14)	0.11292 (13)	0.0241 (4)	
H1C	0.3904	1.0354	0.1140	0.029*	
C2C	0.33983 (14)	0.92483 (14)	0.09105 (12)	0.0227 (4)	
C3C	0.36369 (14)	0.83356 (14)	0.09052 (13)	0.0232 (4)	
C4C	0.44987 (14)	0.78914 (15)	0.10945 (13)	0.0246 (4)	
C5C	0.51171 (14)	0.84139 (15)	0.13116 (13)	0.0241 (4)	
C6C	0.49252 (14)	0.93202 (14)	0.13327 (13)	0.0240 (4)	
C7C	0.47466 (16)	0.69088 (16)	0.10745 (16)	0.0317 (5)	
H7C1	0.4156	0.6587	0.1053	0.048*	
H7C2	0.4923	0.6459	0.1655	0.048*	
H7C3	0.5322	0.7041	0.0503	0.048*	
C8C	0.24356 (14)	0.96932 (14)	0.07192 (13)	0.0241 (4)	
H8C1	0.2544	1.0439	0.0441	0.029*	
H8C2	0.2276	0.9529	0.0247	0.029*	
C9C	0.16854 (14)	0.63680 (15)	0.09894 (14)	0.0251 (4)	
C10C	0.17014 (16)	0.53322 (16)	0.14934 (16)	0.0331 (5)	
H10C	0.1867	0.5055	0.2078	0.040*	
C11C	0.14744 (17)	0.47044 (18)	0.11403 (19)	0.0418 (6)	
H11C	0.1495	0.3996	0.1478	0.050*	
C12C	0.12199 (17)	0.5105 (2)	0.0301 (2)	0.0457 (6)	
H12C	0.1054	0.4670	0.0067	0.055*	
C13C	0.12048 (19)	0.6130 (2)	−0.02015 (19)	0.0462 (6)	
H13C	0.1030	0.6400	−0.0781	0.055*	
C14C	0.14423 (17)	0.67743 (18)	0.01321 (16)	0.0368 (5)	
H14C	0.1439	0.7484	−0.0220	0.044*	
C1D	0.13485 (14)	0.97464 (14)	0.22290 (13)	0.0239 (4)	
H1D	0.1751	1.0356	0.2027	0.029*	
C2D	0.05730 (14)	0.93516 (14)	0.31036 (13)	0.0231 (4)	
C3D	−0.00030 (14)	0.84668 (15)	0.33686 (13)	0.0242 (4)	
C4D	0.01450 (14)	0.79593 (15)	0.28118 (14)	0.0255 (4)	
C5D	0.09407 (14)	0.83894 (14)	0.19564 (13)	0.0232 (4)	
C6D	0.15514 (14)	0.92744 (14)	0.16438 (13)	0.0228 (4)	
C7D	−0.05138 (17)	0.70142 (17)	0.31159 (16)	0.0365 (5)	
H7D1	−0.0530	0.6961	0.2544	0.055*	
H7D2	−0.1204	0.7041	0.3481	0.055*	
H7D3	−0.0237	0.6424	0.3521	0.055*	
C8D	0.03903 (14)	0.98661 (14)	0.37464 (13)	0.0235 (4)	
H8D1	0.0594	1.0602	0.3342	0.028*	
H8D2	−0.0343	0.9763	0.4101	0.028*	
C9D	−0.18126 (14)	0.67358 (15)	0.60172 (14)	0.0258 (4)	
C10D	−0.21742 (16)	0.59045 (16)	0.59617 (15)	0.0319 (5)	
H10D	−0.1763	0.5680	0.5530	0.038*	
C11D	−0.31361 (16)	0.54133 (16)	0.65406 (16)	0.0367 (5)	
H11D	−0.3388	0.4852	0.6505	0.044*	
C12D	−0.37320 (16)	0.57426 (17)	0.71741 (16)	0.0386 (5)	
H12D	−0.4393	0.5406	0.7567	0.046*	
C13D	−0.33707 (16)	0.65558 (18)	0.72374 (16)	0.0388 (5)	
H13D	−0.3781	0.6770	0.7678	0.047*	
C14D	−0.24110 (16)	0.70591 (16)	0.66583 (15)	0.0321 (5)	
H14D	−0.2163	0.7620	0.6698	0.039*	

### Atomic displacement parameters (Å^2^)

**Table:**

	*U*^11^	*U*^22^	*U*^33^	*U*^12^	*U*^13^	*U*^23^
N1	0.026 (2)	0.068 (3)	0.030 (2)	0.002 (2)	−0.0062 (16)	−0.031 (2)
O1	0.0231 (15)	0.0457 (19)	0.0267 (16)	−0.0007 (14)	−0.0038 (12)	−0.0216 (15)
N2	0.025 (2)	0.084 (4)	0.036 (3)	0.012 (2)	−0.0103 (18)	−0.041 (3)
O2	0.0266 (16)	0.0343 (17)	0.0239 (16)	0.0087 (14)	−0.0083 (12)	−0.0176 (15)
B1	0.0572 (18)	0.0266 (13)	0.0331 (14)	0.0055 (12)	−0.0109 (13)	−0.0106 (11)
F1	0.1356 (18)	0.0472 (10)	0.0887 (13)	0.0254 (11)	−0.0712 (13)	−0.0180 (10)
F2	0.0914 (12)	0.0594 (10)	0.0434 (9)	−0.0021 (9)	−0.0133 (8)	−0.0296 (8)
F3	0.0762 (11)	0.0290 (7)	0.0436 (8)	−0.0007 (7)	−0.0064 (7)	−0.0092 (6)
F4	0.0738 (12)	0.0646 (11)	0.0825 (13)	−0.0121 (9)	0.0162 (10)	−0.0432 (10)
P1A	0.0246 (3)	0.0281 (3)	0.0190 (2)	−0.0001 (2)	−0.0069 (2)	−0.0100 (2)
P1B	0.0213 (2)	0.0333 (3)	0.0237 (3)	0.0047 (2)	−0.0060 (2)	−0.0135 (2)
P1C	0.0246 (3)	0.0281 (3)	0.0211 (2)	0.0035 (2)	−0.0079 (2)	−0.0136 (2)
P1D	0.0200 (2)	0.0283 (3)	0.0246 (3)	0.00258 (19)	−0.0042 (2)	−0.0137 (2)
O1A	0.0255 (7)	0.0335 (8)	0.0207 (7)	0.0036 (6)	−0.0088 (6)	−0.0119 (6)
O2A	0.0254 (7)	0.0322 (7)	0.0175 (6)	−0.0013 (6)	−0.0063 (5)	−0.0100 (6)
O3A	0.0361 (8)	0.0342 (8)	0.0264 (7)	−0.0018 (6)	−0.0103 (6)	−0.0151 (6)
O1B	0.0234 (7)	0.0387 (8)	0.0259 (7)	0.0058 (6)	−0.0076 (6)	−0.0170 (6)
O2B	0.0219 (7)	0.0378 (8)	0.0237 (7)	0.0060 (6)	−0.0073 (6)	−0.0150 (6)
O3B	0.0269 (8)	0.0400 (9)	0.0297 (8)	0.0012 (6)	−0.0030 (6)	−0.0139 (7)
O1C	0.0237 (7)	0.0313 (7)	0.0241 (7)	0.0020 (6)	−0.0074 (5)	−0.0161 (6)
O2C	0.0258 (7)	0.0333 (7)	0.0261 (7)	0.0062 (6)	−0.0090 (6)	−0.0193 (6)
O3C	0.0372 (8)	0.0351 (8)	0.0227 (7)	0.0052 (6)	−0.0116 (6)	−0.0136 (6)
O1D	0.0211 (7)	0.0349 (8)	0.0232 (7)	0.0012 (6)	−0.0036 (5)	−0.0149 (6)
O2D	0.0198 (6)	0.0314 (7)	0.0241 (7)	0.0003 (5)	−0.0042 (5)	−0.0143 (6)
O3D	0.0259 (7)	0.0353 (8)	0.0372 (8)	0.0081 (6)	−0.0053 (6)	−0.0190 (7)
C1A	0.0292 (10)	0.0212 (9)	0.0206 (9)	0.0029 (8)	−0.0048 (8)	−0.0107 (8)
C2A	0.0234 (9)	0.0245 (10)	0.0243 (10)	0.0027 (7)	−0.0053 (8)	−0.0143 (8)
C3A	0.0238 (10)	0.0295 (10)	0.0215 (9)	0.0051 (8)	−0.0089 (8)	−0.0133 (8)
C4A	0.0227 (9)	0.0280 (10)	0.0220 (9)	0.0019 (8)	−0.0044 (8)	−0.0121 (8)
C5A	0.0190 (9)	0.0285 (10)	0.0245 (10)	0.0017 (7)	−0.0038 (7)	−0.0152 (8)
C6A	0.0260 (10)	0.0237 (9)	0.0234 (9)	0.0071 (8)	−0.0084 (8)	−0.0150 (8)
C7A	0.0294 (11)	0.0365 (12)	0.0269 (11)	−0.0024 (9)	−0.0079 (9)	−0.0058 (9)
C8A	0.0268 (10)	0.0260 (10)	0.0242 (10)	−0.0005 (8)	−0.0072 (8)	−0.0127 (8)
C9A	0.0207 (9)	0.0348 (11)	0.0206 (9)	0.0016 (8)	−0.0049 (8)	−0.0107 (8)
C10A	0.0332 (11)	0.0338 (11)	0.0283 (11)	0.0051 (9)	−0.0065 (9)	−0.0112 (9)
C11A	0.0400 (13)	0.0418 (13)	0.0295 (12)	0.0120 (11)	−0.0036 (10)	−0.0030 (10)
C12A	0.0374 (13)	0.0701 (18)	0.0227 (11)	0.0156 (12)	−0.0118 (10)	−0.0132 (12)
C13A	0.0482 (15)	0.0689 (18)	0.0307 (12)	0.0020 (13)	−0.0183 (11)	−0.0237 (13)
C14A	0.0420 (13)	0.0430 (13)	0.0264 (11)	−0.0031 (10)	−0.0121 (10)	−0.0129 (10)
C1B	0.0251 (10)	0.0230 (9)	0.0226 (9)	−0.0024 (7)	−0.0081 (8)	−0.0075 (8)
C2B	0.0227 (9)	0.0259 (10)	0.0211 (9)	−0.0051 (7)	−0.0048 (8)	−0.0092 (8)
C3B	0.0194 (9)	0.0331 (11)	0.0229 (10)	0.0016 (8)	−0.0048 (8)	−0.0142 (8)
C4B	0.0234 (10)	0.0331 (11)	0.0243 (10)	0.0020 (8)	−0.0095 (8)	−0.0134 (9)
C5B	0.0239 (9)	0.0310 (10)	0.0171 (9)	−0.0041 (8)	−0.0050 (7)	−0.0100 (8)
C6B	0.0214 (9)	0.0242 (10)	0.0235 (9)	−0.0032 (7)	−0.0063 (8)	−0.0106 (8)
C7B	0.0389 (12)	0.0436 (13)	0.0253 (11)	0.0145 (10)	−0.0136 (9)	−0.0128 (10)
C8B	0.0277 (10)	0.0282 (10)	0.0194 (9)	−0.0032 (8)	−0.0052 (8)	−0.0079 (8)
C9B	0.0232 (10)	0.0376 (11)	0.0345 (11)	0.0060 (8)	−0.0112 (9)	−0.0207 (10)
C10B	0.0312 (11)	0.0327 (12)	0.0453 (13)	0.0029 (9)	−0.0123 (10)	−0.0155 (10)
C11B	0.0396 (13)	0.0287 (12)	0.0712 (18)	0.0076 (10)	−0.0239 (13)	−0.0206 (12)
C12B	0.0305 (12)	0.0490 (15)	0.081 (2)	0.0129 (11)	−0.0206 (13)	−0.0453 (15)
C13B	0.0350 (13)	0.0731 (19)	0.0529 (16)	0.0167 (13)	−0.0103 (12)	−0.0414 (15)
C14B	0.0358 (13)	0.0615 (16)	0.0351 (12)	0.0169 (11)	−0.0117 (10)	−0.0243 (12)
C1C	0.0290 (10)	0.0238 (9)	0.0155 (9)	0.0007 (8)	−0.0039 (8)	−0.0074 (7)
C2C	0.0221 (9)	0.0264 (10)	0.0136 (8)	0.0009 (7)	−0.0020 (7)	−0.0064 (7)
C3C	0.0231 (9)	0.0285 (10)	0.0174 (9)	−0.0005 (8)	−0.0053 (7)	−0.0104 (8)
C4C	0.0244 (10)	0.0279 (10)	0.0185 (9)	0.0014 (8)	−0.0032 (7)	−0.0102 (8)
C5C	0.0200 (9)	0.0311 (10)	0.0178 (9)	0.0029 (8)	−0.0045 (7)	−0.0091 (8)
C6C	0.0254 (10)	0.0269 (10)	0.0140 (8)	−0.0035 (8)	−0.0029 (7)	−0.0061 (8)
C7C	0.0295 (11)	0.0348 (11)	0.0355 (11)	0.0076 (9)	−0.0103 (9)	−0.0201 (10)
C8C	0.0261 (10)	0.0251 (10)	0.0188 (9)	0.0033 (8)	−0.0062 (8)	−0.0086 (8)
C9C	0.0220 (9)	0.0308 (10)	0.0269 (10)	0.0032 (8)	−0.0063 (8)	−0.0179 (9)
C10C	0.0302 (11)	0.0330 (11)	0.0360 (12)	0.0052 (9)	−0.0082 (9)	−0.0172 (10)
C11C	0.0346 (12)	0.0349 (12)	0.0553 (15)	0.0012 (10)	−0.0030 (11)	−0.0266 (12)
C12C	0.0331 (12)	0.0603 (17)	0.0659 (17)	0.0036 (11)	−0.0112 (12)	−0.0499 (15)
C13C	0.0460 (14)	0.0673 (18)	0.0474 (14)	0.0100 (12)	−0.0237 (12)	−0.0396 (14)
C14C	0.0420 (13)	0.0415 (13)	0.0356 (12)	0.0095 (10)	−0.0180 (10)	−0.0218 (10)
C1D	0.0249 (10)	0.0225 (9)	0.0236 (10)	0.0046 (7)	−0.0092 (8)	−0.0092 (8)
C2D	0.0243 (9)	0.0256 (10)	0.0231 (9)	0.0078 (8)	−0.0102 (8)	−0.0129 (8)
C3D	0.0200 (9)	0.0319 (10)	0.0204 (9)	0.0037 (8)	−0.0050 (7)	−0.0125 (8)
C4D	0.0227 (9)	0.0314 (10)	0.0262 (10)	0.0034 (8)	−0.0081 (8)	−0.0161 (9)
C5D	0.0235 (9)	0.0302 (10)	0.0229 (9)	0.0078 (8)	−0.0093 (8)	−0.0171 (8)
C6D	0.0227 (9)	0.0258 (10)	0.0201 (9)	0.0067 (7)	−0.0089 (7)	−0.0096 (8)
C7D	0.0336 (12)	0.0433 (13)	0.0365 (12)	−0.0068 (10)	−0.0026 (10)	−0.0258 (11)
C8D	0.0250 (10)	0.0243 (9)	0.0232 (9)	0.0067 (8)	−0.0077 (8)	−0.0126 (8)
C9D	0.0212 (9)	0.0282 (10)	0.0238 (10)	0.0027 (8)	−0.0066 (8)	−0.0088 (8)
C10D	0.0305 (11)	0.0316 (11)	0.0324 (11)	0.0034 (9)	−0.0093 (9)	−0.0139 (9)
C11D	0.0326 (12)	0.0286 (11)	0.0417 (13)	−0.0010 (9)	−0.0120 (10)	−0.0095 (10)
C12D	0.0242 (11)	0.0371 (12)	0.0360 (12)	0.0006 (9)	−0.0053 (9)	−0.0035 (10)
C13D	0.0289 (11)	0.0463 (14)	0.0326 (12)	0.0053 (10)	−0.0007 (9)	−0.0165 (11)
C14D	0.0289 (11)	0.0363 (12)	0.0296 (11)	0.0034 (9)	−0.0048 (9)	−0.0163 (9)

### Geometric parameters (Å, º)

**Table:**

N1—O1	0.966 (5)	C7B—H7B2	0.9800
N2—O2	0.885 (5)	C7B—H7B3	0.9800
B1—F3	1.374 (3)	C8B—C6C	1.524 (3)
B1—F2	1.379 (3)	C8B—H8B1	0.9900
B1—F1	1.382 (3)	C8B—H8B2	0.9900
B1—F4	1.387 (3)	C9B—C14B	1.392 (3)
P1A—O3A	1.4716 (14)	C9B—C10B	1.397 (3)
P1A—O1A	1.5894 (14)	C10B—C11B	1.397 (3)
P1A—O2A	1.5950 (14)	C10B—H10B	0.9500
P1A—C9A	1.768 (2)	C11B—C12B	1.374 (4)
P1B—O3B	1.4688 (15)	C11B—H11B	0.9500
P1B—O2B	1.5825 (14)	C12B—C13B	1.366 (4)
P1B—O1B	1.5943 (15)	C12B—H12B	0.9500
P1B—C9B	1.776 (2)	C13B—C14B	1.392 (3)
P1C—O3C	1.4695 (14)	C13B—H13B	0.9500
P1C—O1C	1.5911 (14)	C14B—H14B	0.9500
P1C—O2C	1.5921 (14)	C1C—C2C	1.391 (3)
P1C—C9C	1.7727 (19)	C1C—C6C	1.396 (3)
P1D—O3D	1.4738 (14)	C1C—H1C	0.9500
P1D—O2D	1.5867 (14)	C2C—C3C	1.393 (3)
P1D—O1D	1.5916 (14)	C2C—C8C	1.521 (3)
P1D—C9D	1.7708 (19)	C3C—C4C	1.392 (3)
O1A—C3A	1.420 (2)	C4C—C5C	1.392 (3)
O2A—C5B	1.418 (2)	C4C—C7C	1.506 (3)
O1B—C3B	1.425 (2)	C5C—C6C	1.382 (3)
O2B—C5C	1.412 (2)	C7C—H7C1	0.9800
O1C—C3C	1.418 (2)	C7C—H7C2	0.9800
O2C—C5D	1.418 (2)	C7C—H7C3	0.9800
O1D—C3D	1.422 (2)	C8C—C6D	1.518 (3)
O2D—C5A	1.418 (2)	C8C—H8C1	0.9900
C1A—C2A	1.392 (3)	C8C—H8C2	0.9900
C1A—C6A	1.399 (3)	C9C—C10C	1.388 (3)
C1A—H1A	0.9500	C9C—C14C	1.394 (3)
C2A—C3A	1.389 (3)	C10C—C11C	1.386 (3)
C2A—C8A	1.520 (3)	C10C—H10C	0.9500
C3A—C4A	1.397 (3)	C11C—C12C	1.377 (4)
C4A—C5A	1.391 (3)	C11C—H11C	0.9500
C4A—C7A	1.502 (3)	C12C—C13C	1.374 (4)
C5A—C6A	1.388 (3)	C12C—H12C	0.9500
C6A—C8D	1.519 (3)	C13C—C14C	1.387 (3)
C7A—H7A1	0.9800	C13C—H13C	0.9500
C7A—H7A2	0.9800	C14C—H14C	0.9500
C7A—H7A3	0.9800	C1D—C6D	1.389 (3)
C8A—C6B	1.521 (3)	C1D—C2D	1.393 (3)
C8A—H8A1	0.9900	C1D—H1D	0.9500
C8A—H8A2	0.9900	C2D—C3D	1.385 (3)
C9A—C14A	1.385 (3)	C2D—C8D	1.522 (3)
C9A—C10A	1.396 (3)	C3D—C4D	1.395 (3)
C10A—C11A	1.391 (3)	C4D—C5D	1.389 (3)
C10A—H10A	0.9500	C4D—C7D	1.502 (3)
C11A—C12A	1.370 (4)	C5D—C6D	1.391 (3)
C11A—H11A	0.9500	C7D—H7D1	0.9800
C12A—C13A	1.380 (4)	C7D—H7D2	0.9800
C12A—H12A	0.9500	C7D—H7D3	0.9800
C13A—C14A	1.391 (3)	C8D—H8D1	0.9900
C13A—H13A	0.9500	C8D—H8D2	0.9900
C14A—H14A	0.9500	C9D—C14D	1.394 (3)
C1B—C6B	1.391 (3)	C9D—C10D	1.402 (3)
C1B—C2B	1.398 (3)	C10D—C11D	1.385 (3)
C1B—H1B	0.9500	C10D—H10D	0.9500
C2B—C3B	1.392 (3)	C11D—C12D	1.390 (3)
C2B—C8B	1.515 (3)	C11D—H11D	0.9500
C3B—C4B	1.392 (3)	C12D—C13D	1.384 (3)
C4B—C5B	1.396 (3)	C12D—H12D	0.9500
C4B—C7B	1.498 (3)	C13D—C14D	1.387 (3)
C5B—C6B	1.391 (3)	C13D—H13D	0.9500
C7B—H7B1	0.9800	C14D—H14D	0.9500
			
F3—B1—F2	111.3 (2)	H8B1—C8B—H8B2	107.9
F3—B1—F1	109.5 (2)	C14B—C9B—C10B	119.8 (2)
F2—B1—F1	108.9 (2)	C14B—C9B—P1B	121.19 (17)
F3—B1—F4	110.4 (2)	C10B—C9B—P1B	118.96 (16)
F2—B1—F4	110.1 (2)	C11B—C10B—C9B	119.5 (2)
F1—B1—F4	106.6 (2)	C11B—C10B—H10B	120.3
O3A—P1A—O1A	113.25 (8)	C9B—C10B—H10B	120.3
O3A—P1A—O2A	113.39 (8)	C12B—C11B—C10B	120.1 (2)
O1A—P1A—O2A	105.21 (7)	C12B—C11B—H11B	119.9
O3A—P1A—C9A	116.42 (9)	C10B—C11B—H11B	119.9
O1A—P1A—C9A	103.86 (8)	C13B—C12B—C11B	120.5 (2)
O2A—P1A—C9A	103.48 (8)	C13B—C12B—H12B	119.7
O3B—P1B—O2B	114.65 (8)	C11B—C12B—H12B	119.7
O3B—P1B—O1B	112.61 (8)	C12B—C13B—C14B	120.8 (2)
O2B—P1B—O1B	105.36 (8)	C12B—C13B—H13B	119.6
O3B—P1B—C9B	116.20 (10)	C14B—C13B—H13B	119.6
O2B—P1B—C9B	102.10 (9)	C9B—C14B—C13B	119.3 (2)
O1B—P1B—C9B	104.63 (8)	C9B—C14B—H14B	120.3
O3C—P1C—O1C	113.22 (8)	C13B—C14B—H14B	120.3
O3C—P1C—O2C	113.28 (8)	C2C—C1C—C6C	121.17 (18)
O1C—P1C—O2C	105.45 (7)	C2C—C1C—H1C	119.4
O3C—P1C—C9C	116.01 (9)	C6C—C1C—H1C	119.4
O1C—P1C—C9C	103.90 (8)	C1C—C2C—C3C	117.94 (17)
O2C—P1C—C9C	103.83 (8)	C1C—C2C—C8C	120.41 (17)
O3D—P1D—O2D	113.92 (8)	C3C—C2C—C8C	121.62 (17)
O3D—P1D—O1D	112.87 (8)	C4C—C3C—C2C	123.55 (17)
O2D—P1D—O1D	105.77 (7)	C4C—C3C—O1C	117.27 (16)
O3D—P1D—C9D	116.93 (9)	C2C—C3C—O1C	119.14 (16)
O2D—P1D—C9D	103.46 (8)	C5C—C4C—C3C	115.43 (17)
O1D—P1D—C9D	102.56 (8)	C5C—C4C—C7C	121.96 (18)
C3A—O1A—P1A	120.78 (12)	C3C—C4C—C7C	122.60 (18)
C5B—O2A—P1A	118.31 (11)	C6C—C5C—C4C	124.08 (18)
C3B—O1B—P1B	119.32 (12)	C6C—C5C—O2B	118.99 (17)
C5C—O2B—P1B	121.91 (11)	C4C—C5C—O2B	116.88 (17)
C3C—O1C—P1C	120.00 (11)	C5C—C6C—C1C	117.82 (17)
C5D—O2C—P1C	118.16 (11)	C5C—C6C—C8B	121.32 (18)
C3D—O1D—P1D	119.51 (12)	C1C—C6C—C8B	120.85 (17)
C5A—O2D—P1D	121.38 (11)	C4C—C7C—H7C1	109.5
C2A—C1A—C6A	122.21 (17)	C4C—C7C—H7C2	109.5
C2A—C1A—H1A	118.9	H7C1—C7C—H7C2	109.5
C6A—C1A—H1A	118.9	C4C—C7C—H7C3	109.5
C3A—C2A—C1A	117.45 (17)	H7C1—C7C—H7C3	109.5
C3A—C2A—C8A	121.68 (17)	H7C2—C7C—H7C3	109.5
C1A—C2A—C8A	120.82 (17)	C6D—C8C—C2C	110.63 (15)
C2A—C3A—C4A	123.67 (18)	C6D—C8C—H8C1	109.5
C2A—C3A—O1A	118.98 (16)	C2C—C8C—H8C1	109.5
C4A—C3A—O1A	117.30 (16)	C6D—C8C—H8C2	109.5
C5A—C4A—C3A	115.46 (17)	C2C—C8C—H8C2	109.5
C5A—C4A—C7A	121.91 (17)	H8C1—C8C—H8C2	108.1
C3A—C4A—C7A	122.63 (18)	C10C—C9C—C14C	120.11 (19)
C6A—C5A—C4A	124.41 (17)	C10C—C9C—P1C	118.53 (16)
C6A—C5A—O2D	118.99 (17)	C14C—C9C—P1C	121.36 (16)
C4A—C5A—O2D	116.51 (16)	C11C—C10C—C9C	119.7 (2)
C5A—C6A—C1A	116.79 (17)	C11C—C10C—H10C	120.2
C5A—C6A—C8D	122.56 (17)	C9C—C10C—H10C	120.2
C1A—C6A—C8D	120.65 (17)	C12C—C11C—C10C	120.1 (2)
C4A—C7A—H7A1	109.5	C12C—C11C—H11C	119.9
C4A—C7A—H7A2	109.5	C10C—C11C—H11C	119.9
H7A1—C7A—H7A2	109.5	C13C—C12C—C11C	120.4 (2)
C4A—C7A—H7A3	109.5	C13C—C12C—H12C	119.8
H7A1—C7A—H7A3	109.5	C11C—C12C—H12C	119.8
H7A2—C7A—H7A3	109.5	C12C—C13C—C14C	120.5 (2)
C2A—C8A—C6B	111.10 (15)	C12C—C13C—H13C	119.8
C2A—C8A—H8A1	109.4	C14C—C13C—H13C	119.8
C6B—C8A—H8A1	109.4	C13C—C14C—C9C	119.2 (2)
C2A—C8A—H8A2	109.4	C13C—C14C—H14C	120.4
C6B—C8A—H8A2	109.4	C9C—C14C—H14C	120.4
H8A1—C8A—H8A2	108.0	C6D—C1D—C2D	121.92 (18)
C14A—C9A—C10A	120.16 (19)	C6D—C1D—H1D	119.0
C14A—C9A—P1A	121.45 (16)	C2D—C1D—H1D	119.0
C10A—C9A—P1A	118.38 (16)	C3D—C2D—C1D	117.33 (17)
C11A—C10A—C9A	119.4 (2)	C3D—C2D—C8D	121.87 (17)
C11A—C10A—H10A	120.3	C1D—C2D—C8D	120.79 (17)
C9A—C10A—H10A	120.3	C2D—C3D—C4D	124.00 (17)
C12A—C11A—C10A	120.4 (2)	C2D—C3D—O1D	119.04 (16)
C12A—C11A—H11A	119.8	C4D—C3D—O1D	116.93 (17)
C10A—C11A—H11A	119.8	C5D—C4D—C3D	115.41 (17)
C11A—C12A—C13A	120.4 (2)	C5D—C4D—C7D	122.33 (17)
C11A—C12A—H12A	119.8	C3D—C4D—C7D	122.26 (18)
C13A—C12A—H12A	119.8	C4D—C5D—C6D	123.81 (17)
C12A—C13A—C14A	120.2 (2)	C4D—C5D—O2C	116.92 (16)
C12A—C13A—H13A	119.9	C6D—C5D—O2C	119.27 (16)
C14A—C13A—H13A	119.9	C1D—C6D—C5D	117.52 (17)
C9A—C14A—C13A	119.5 (2)	C1D—C6D—C8C	120.72 (17)
C9A—C14A—H14A	120.3	C5D—C6D—C8C	121.72 (17)
C13A—C14A—H14A	120.3	C4D—C7D—H7D1	109.5
C6B—C1B—C2B	122.22 (18)	C4D—C7D—H7D2	109.5
C6B—C1B—H1B	118.9	H7D1—C7D—H7D2	109.5
C2B—C1B—H1B	118.9	C4D—C7D—H7D3	109.5
C3B—C2B—C1B	117.18 (17)	H7D1—C7D—H7D3	109.5
C3B—C2B—C8B	122.31 (18)	H7D2—C7D—H7D3	109.5
C1B—C2B—C8B	120.48 (18)	C6A—C8D—C2D	111.24 (15)
C4B—C3B—C2B	123.83 (18)	C6A—C8D—H8D1	109.4
C4B—C3B—O1B	116.91 (17)	C2D—C8D—H8D1	109.4
C2B—C3B—O1B	119.24 (16)	C6A—C8D—H8D2	109.4
C3B—C4B—C5B	115.61 (18)	C2D—C8D—H8D2	109.4
C3B—C4B—C7B	122.11 (18)	H8D1—C8D—H8D2	108.0
C5B—C4B—C7B	122.28 (18)	C14D—C9D—C10D	120.33 (18)
C6B—C5B—C4B	123.92 (17)	C14D—C9D—P1D	121.92 (16)
C6B—C5B—O2A	119.10 (17)	C10D—C9D—P1D	117.74 (15)
C4B—C5B—O2A	116.97 (17)	C11D—C10D—C9D	119.5 (2)
C5B—C6B—C1B	117.21 (18)	C11D—C10D—H10D	120.3
C5B—C6B—C8A	122.02 (17)	C9D—C10D—H10D	120.3
C1B—C6B—C8A	120.71 (17)	C10D—C11D—C12D	119.9 (2)
C4B—C7B—H7B1	109.5	C10D—C11D—H11D	120.0
C4B—C7B—H7B2	109.5	C12D—C11D—H11D	120.0
H7B1—C7B—H7B2	109.5	C13D—C12D—C11D	120.6 (2)
C4B—C7B—H7B3	109.5	C13D—C12D—H12D	119.7
H7B1—C7B—H7B3	109.5	C11D—C12D—H12D	119.7
H7B2—C7B—H7B3	109.5	C12D—C13D—C14D	120.1 (2)
C2B—C8B—C6C	112.07 (15)	C12D—C13D—H13D	119.9
C2B—C8B—H8B1	109.2	C14D—C13D—H13D	119.9
C6C—C8B—H8B1	109.2	C13D—C14D—C9D	119.5 (2)
C2B—C8B—H8B2	109.2	C13D—C14D—H14D	120.2
C6C—C8B—H8B2	109.2	C9D—C14D—H14D	120.2

### Hydrogen-bond geometry (Å, º)

*Cg*1, *Cg*2 and *Cg*3 are the centroids of the aromatic rings C9*B*–C14*B*, C9*D*–C14*D* and C1*A*–C6*A*, respectively.

**Table:**

*D*—H···*A*	*D*—H	H···*A*	*D*···*A*	*D*—H···*A*
C1*B*^i^—H1*B*^i^···F1	0.95	2.41	3.344 (3)	169
C14*B*^ii^—H14*B*^ii^···F2	0.95	2.57	3.357 (3)	140
C7*C*^ii^—H7*C*3^ii^···F2	0.98	2.62	3.484 (2)	147
C8*C*^i^—H8*C*1^i^···F2	0.98	2.49	3.379 (3)	150
C1*D*^i^—H1*D*^i^···F2	0.95	2.60	3.439 (2)	147
C11*A*^iii^—H11*A*^iii^···F3	0.95	2.45	3.254 (2)	142
C7*C*^ii^—H7*C*3^ii^···F3	0.98	2.64	3.569 (3)	160
C11*C*—H11*C*···F4	0.95	2.53	3.447 (3)	162
C1*D*^i^—H1*D*^i^···F4	0.95	2.65	3.509 (3)	150
C14*D*^iv^—H14*D*^iv^···F4	0.95	2.63	3.336 (4)	131
C7*D*—H7*D*1···*Cg*1^v^	0.98	2.80	3.524 (4)	131
C7*B*—H7*B*1···*Cg*2^vi^	0.98	2.88	3.530 (4)	124
C8*D*—H8*D*2···*Cg*3^vii^	0.98	2.87	3.594 (3)	131

Symmetry codes: (i) *x*, *y*−1, *z*; (ii) −*x*+1, −*y*+1, −*z*; (iii) *x*, *y*, *z*−1; (iv) −*x*, −*y*+1, −*z*+1; (v) *x*−1, *y*, *z*; (vi) *x*+1, *y*, *z*; (vii) −*x*, −*y*+2, −*z*+1.
