# Supplementary figures and images for: Nitro­sonium complexation by the tetra­phospho­nate cavitand 5,11,17,23-tetra­methyl-6,10:12,16:18,22:24,4-tetra­kis­(phenyl­phospho­nato-κ2 O,O)resorcin(4)arene
Source: Acta Crystallogr E Crystallogr Commun. 2017 Nov 3;73(Pt 12):1801–5. doi: 10.1107/S2056989017015857 (PMC5730227; doi:10.1107/S2056989017015857)

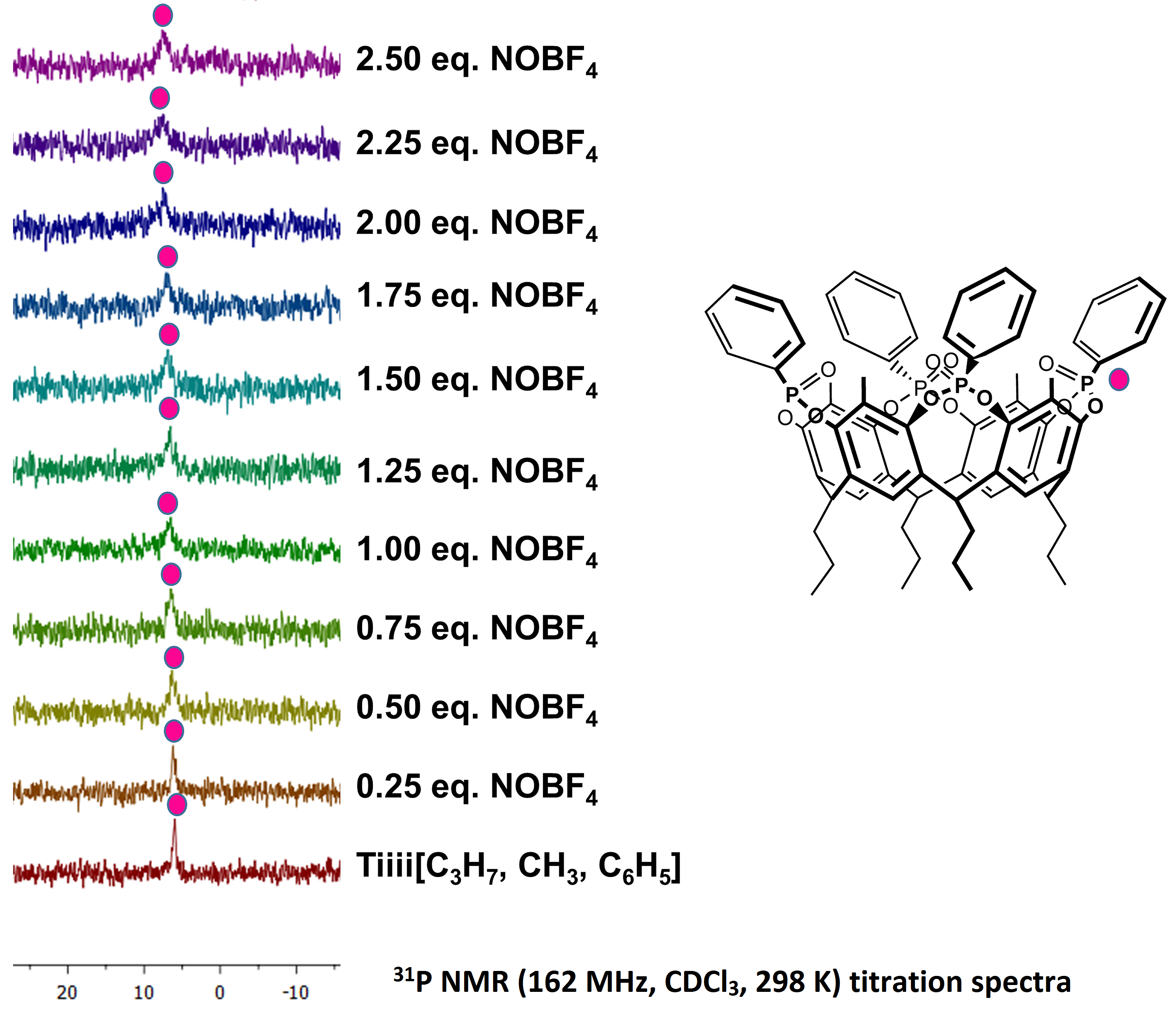

Supplement: Supplementary file 3 [file e-73-01801-sup3.tif]
